# Mixed epithelial and stromal tumor of the kidney: report of eight cases and literature review

**DOI:** 10.1186/1477-7819-11-207

**Published:** 2013-08-20

**Authors:** Chao-jun Wang, Yi-wei Lin, Hua Xiang, Dan-bo Fang, Peng Jiang, Bo-hua Shen

**Affiliations:** 1 Department of Urology, The First Affiliated Hospital, School of Medicine, Zhejiang University, Qingchun Road 79, 310003 Hangzhou, Zhejiang Province, China; 2 Department of Pathology, The First Affiliated Hospital, School of Medicine, Zhejiang University, Hangzhou, Zhejiang Province, China

**Keywords:** Kidney, Mixed epithelial and stromal tumor

## Abstract

Mixed epithelial and stromal tumor of the kidney (MESTK) is the term given to a class of uncommon biphasic tumors of the kidney, with few reported cases. We describe eight cases of MESTK with detailed clinicopathological data and follow-up information. With this report, we hope to increase clinical awareness that MESTK should be considered as one of the possible diagnoses for cystic renal mass, especially in peri-menopausal women or those who receive hormone therapy. In addition, regular follow-up is necessary for the any cases with malignant potential.

## Background

Mixed epithelial and stromal tumor of the kidney (MESTK), is a rare kidney tumor [1]. The tumor was first identified by Michal and Syrucek in 1998 [2] and has been ariously termed ‘cystic hamartoma of the renal pelvis,’ ‘adult mesoblastic nephroma,’ ‘cystic nephroma,’ ‘mature nephroblastic tumor’ or ‘cystic partially differentiated nephroblastoma.’ To date, approximately 100 cases have been reported [3], with most of these reports focusing on the pathological and radiological features of the tumors. In this paper, we report detailed clinicopathological findings and clinical outcomes of a series of MESTK cases, and review the related literature.

## Case presentation

During the period 2005 to 2012, eight cases with a diagnosis of MESTK were identified from the surgical pathology files of the urology department at our hospital. The clinical information and pathological data were obtained from the medical records, and demographic information, presenting symptoms, treatment, tumor size, immunohistochemical staining profiles, and scheduled follow-up data were collected.

**Figure 1:**
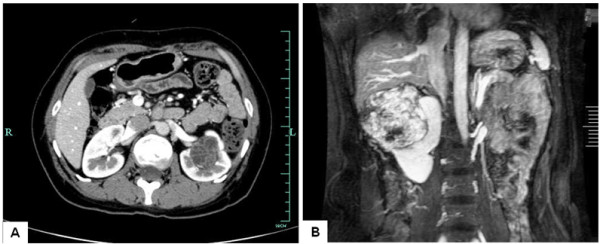
**Representative radiological findings of mixed epithelial and stromal tumor of the kidney. (A)** Patient 3. Abdominal computed tomography scan showed a left renal tumor with cystic and solid components. **(B)** Patient 7. T2-weighted coronal magnetic resonance imaging showed a giant, well-circumscribed, multi-cystic tumor that had originated from the right kidney.

The clinical features and follow-up data are summarized in Table 1. Of the eight patients, six were women and two were men. Mean age at presentation was 38 years. The initial clinical presentation in one patient was flank pain, but all the other cases were discovered incidentally during regular examination. None of these patients had any history of hormonal therapy. In all cases, the computed tomography (CT) scan showed a partially cystic mass in the kidney, which was classified as a Bosniak III or IV lesion, indicating a pre-operative clinical impression of cystic renal cancer (Figure 1). Thus all eight patients underwent either nephrectomy or partial nephrectomy, and the diagnosis of MESTK was made postoperatively.

**Table 1:** Clinicopathologic features of 8 patients with mixed epithelial and stromal tumor of the kidney

**Patient number**	**Age, years**	**Sex**	**Clinical presentation**	**Treatment**	**Tumor size, cm**	**IHC profile**	**Length of follow-up, months**
1	56	F	Incidental	Nephrectomy	4.5	Postive for ER and PR; negative for CD10	4
2	60	M	Incidental	Nephrectomy	3.5	Positive for CK, PR, SMA, desmin, and vimentin	6
3	40	F	Incidental	Nephrectomy	4	Positive for ER, PR, CK, vimentin, and CD10	19
4	58	M	Incidental	Nephrectomy	4	Positive for CK, CD10, ER, PR, vimentin, and desmin; negative for Melan-A and HMB-45	23
5	54	F	Incidental	Partial Nephrectomy	3.5	Positive for ER and PR; negative for CD10	32
6	47	F	Incidental	Partial Nephrectomy	3.5	Positive for SMA, desmin, CD10, PR; negative for HMB-45	42
7	33	F	Pain	Nephrectomy	9.5	Positive for desmin, CD99, and S-100; negative for HMB-45, SMA, Myo-D1, CD31, and CD3	48
8	51	F	Incidental	Nephrectomy	7	Positive for ER, PR, desmin, and CD10; negative for HMB-45	50

*Abbreviations*: CK, cytokeratin; ER, estrogen receptor; IHC, Immunohistochemistry; PR, progesterone receptor; SMA, smooth muscle actin.

**Figure 2:**
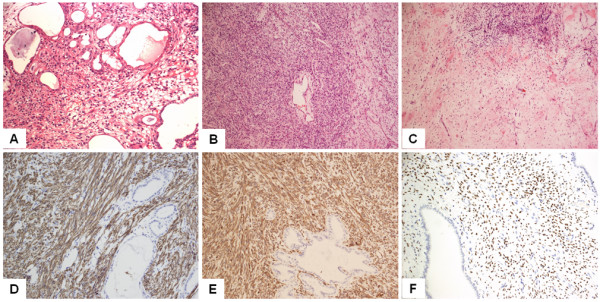
**Representative pathological findings of mixed epithelial and stromal tumor of the kidney. (A)** MESTK showed characteristic biphasic components, including tubules embedded in the spindle cell stroma. **(B)** The mesenchymal component resembled that of densely cellular ovarian stroma. **(C)** The ovarian-like stroma underwent myxoid change. **(D)** Smooth muscle marker such as desmin was strongly positive in the stroma of MESTK. **(E)** The stroma showed a positive reaction against vimentin. **(F)** Progesterone receptors were seen in the nuclei of the stromal cells of MESTK.

On gross examination, the excised specimens were found to be of varying size and consisted of multi-cystic and solid septa. Histological examination showed that all specimens were composed of cysts or dilated tubules of diverse diameter. All specimens presented with the characteristic mixture of epithelial and stromal components (Figure 2A). The tubular glandular epithelium was scattered within abundant spindle cells. Assays showed that the specimens had diverse immunochemical profiles (Table 1).

The patients were followed up for a mean duration of 28 months (4 to 50 months); at the end of which, all eight patients were alive without any evidence of recurrence or metastasis.

## Discussion

MESTK, which was included in the WHO 2004 renal tumor classification, is a rare and distinctive kidney tumor composed of both epithelium and stroma with solid and cystic architecture [4]. As reported previously [5,6], MESTK occurs predominantly in middle-aged peri-menopausal women and older women, especially those with a history of estrogen therapy, which indicates an underlying association between estrogen and MESTK. However, there are some rare male [5,7-9] or pediatric [7,10,11] cases. In our series, most of the female patients were middle-aged peri-menopausal to older women. This implies that a disturbed hormonal environment contributes to the pathogenesis of MESTK; however, we did not observe any correlation between estrogen therapy and MESTK, as all the patients denied histories of hormonal therapy. Thus, whether estrogen therapy is indeed associated with MESTK warrants further study.

The most common clinical presentations of MESTK include palpable abdominal mass, flank pain, and hematuria. However, in our series, almost all the cases were asymptomatic, and wee detected only incidentally during regular investigation. Radiologically, MESTK appears as well-circumscribed multi-septate cystic mass with solid components and thick or thin septa on both CT and magnetic resonance imaging scans, and thus can mimic complex renal cyst or cystic nephroma [12-14]. Lack of any typical radiological features makes it difficult to establish a precise diagnosis of MESTK preoperatively [3], thus, most cases are confirmed postoperatively, as in our series.

Histologically, MESTK is a dimorphic tumor composed of cysts and tubules embedded in the spindle cell stroma. The histogenesis of MESTK is unknown, and it has been proposed that both components of the tumor, stromal and epithelial, are neoplastic [15] and probably arise from a common cell of origin [16]. Miscroscopically, the stroma may resemble ovarian stroma, both morphologically and immunohistochemically (Figure 2B,C), and is composed of clusters of tubules or cystically dilated glands with variable lining [17]. Immunohistochemically, the epithelial components are usually positive for epithelial membrane antigen and cytokeratin. Spindle cells usually show diffusely and strongly positive immunostaining with desmin (Figure 2D), smooth muscle actin, and vimentin (Figure 2E). In addition, there is a high frequency of estrogen and progesterone receptor present in the nuclei of the spindle cells (Figure 2F). The distinctive ER and PR expression pattern seems to support the hypothesis that hormonal hyperstimulation contributes to tumorigenesis of MESTK.

MESTK used to be categorized as a benign renal tumor without recurrence or metastasis. However, malignant transformation of MESTK has been recently recognized, and a small number of aggressive cases with varied transformation have been described in the literature [8,9,18-21]. All of these malignant cases were female patients with the exception of one male patient reported by Suzuki *et al*. [8,22]. The prognosis for malignant MESTK varies from cases to cases, with half of the reported cases considered to have a dismal prognosis. There is no established predictor for prognosis, thus the histogenesis and clinical behavior of MESTK warrants further study.

## Conclusions

MESTK is a rare clinical entity. It is generally considered to be a benign tumor with good prognosis, but there is malignant potential. MESTK should be considered as a possible diagnosis in cases of cystic renal mass, especially in peri-menopausal women or those who have received hormonal therapy.

## Consent

Written informed consent was obtained from the patient for publication of this report and any accompanying images.

## Competing interests

The authors declare that they have no competing interests.

## Authors’ contributions

CW and YL summarized the clinicopathologic data and writed the manuscript; HX provided the pathological information; DF and PJ collected the clinical data, and BS revised and edited the manuscript. All authors read and approved the final manuscript.
